# Identification of urban functions enhancement and weakening based on urban land use conversion: A case study of Changchun, China

**DOI:** 10.1371/journal.pone.0234522

**Published:** 2020-06-24

**Authors:** Guolei Zhou, Chenggu Li, Jing Zhang

**Affiliations:** School of Geographical Sciences, Northeast Normal University, Changchun, Jilin Province, China; Institute for Advanced Sustainability Studies, GERMANY

## Abstract

A deep understanding of urban functions is the basis for the optimization of urban spatial structure and for the sustainable development of urban space. Previous research mainly focused on the identification and classification of urban functions using a variety of data and methods; however, little attention has been paid to the quantitative measurement of changes in urban functions, and to their temporal and spatial evolution. With the help of urban land use maps, we used the overlay analysis and the transition matrix to quantitatively measure the enhancement and weakening of urban functions and their spatial differences, by analyzing the relationship between urban land development and redevelopment. Accordingly, the change state of urban function enhancement and weakening could be divided into six categories. Moreover, the results of research on residential, commercial, public service, and industrial functions show that changes in different urban functions have different temporal and spatial characteristics. Our findings on the enhancement and weakening of urban functions in the central city of Changchun provide theoretical support for the sustainable development of urban space in the future.

## 1. Introduction

Cities are giant, complex systems, composed of different functional areas that involve various types of urban land [1–4], such as residential areas, commercial areas, and industrial areas. Rapid urbanization causes urban spatial expansion and urban renewal, which in turn trigger the diffusion and agglomeration of urban functions and the reorganization of urban functional areas [5–6]. Changes in urban land inevitably cause changes in urban functions. Urban land expansion on the periphery of urban built-up areas entails the outward diffusion of urban functions. Urban land redevelopment within urban built-up areas entails the replacement of one type of urban land with another type, as well as changes in urban functions, which have great implications for urban spatial structure and sustainable urban development [7–8].

Scholars have focused on the interaction between urban function and urban spatial structure. Fuentes (2000) has proven that changes in urban functions in Ciudad Juárez have affected the urban spatial structure [8]. The findings of Tian et al. (2010) showed that the spatial structure of Beijing’s urban functions exhibits characteristics that are in line with the Burgess’s concentric zone theory [9]. Salvati (2019) pointed out that the relationship between urban functions and the distance from the inner city affects the structure of urban spac [10]. Some research proved that the spread of urban functions has led to the transformation of a single-center urban spatial structure into a polycentric, decentralized urban spatial structure[10–14]. The diversity of form and function is also closely related to urban development [15–17]. The relationship between form (i.e., urban spatial structure) and function (i.e., urban function) can reveal the complexity of urban areas, as the diversification of urban functions plays a positive role in shaping urban space [18–21]. Compared with the classic indicators of urban growth, Carlucci et al. (2019) proposed that diversification in urban functions may effectively reflect a new metropolitan geography and the complexity of cities [22]. Urban functions are often intertwined and interrelated in a city [23], forming a highly dynamic and diverse urban landscape [24]. Chen et al. (2020) used ‘Point-of-Interest’ (POI) data to analyze the spatial organization of urban functions in 25 Chinese cities [25].

The identification of urban functions and urban functional areas is another hot topic for scholars. Urban function refers to the actual use of urban space by various human activities [24, 26]. Traditional research normally uses remote sensing images to assess urban land use type and identify urban functional areas [27–31]. In recent years, with the rapid development of the Internet technology, big data has been increasingly used in research on urban functions. Based on spatio-temporal taxi OD trips data and using a non-negative matrix factorization model, Zhou et al. explored how to identify and classify urban functions [1]. Gao et al. (2017) used POI data and human activity data obtained from social media to construct a new framework to study urban functional areas [2]. In Tu et al.’s research, mobile phone and social media data were used to identify the diurnal changes in urban functions [7]. Gao et al. (2019) applied vehicle trajectory data to identify the categories of urban functional areas [32]. Moreover, several scholars performed semantic analyses on the basis of POI data to identify different urban functional divisions [33–36]. In order to avoid subjective errors in the semantic analysis of POI data, Yi et al. used the Fisher’s exact test to identify urban functions and districts [37]. In addition, some scholars have explored the classification of urban functions from a micro-level perspective. Gong et al. used street view images, instead of remote sensing images, to classify street functional spaces from the perspective of citizens [38]. Xing et al. innovatively used spatial metrics to measure urban landscape, to obtain an urban functional classification [39].

Previous research has focused on the identification and classification of urban functions using a variety of data and methods; however, little attention has been paid to the measurement of enhancement or weakening of urban functions and their temporal and spatial evolution. Our research used urban land use maps that have been tracked and mapped for many years, identified the type of urban function through the urban land use type, measured the enhancement and weakening of urban functions through the relationship between development and redevelopment of urban land, and analyzed the temporal and spatial characteristics of urban function changes. It is difficult to measure the enhancement and weakening of urban functions because of its abstraction. In our work, the concept of urban land conversion is proposed, and changes in urban functions are quantitatively measured. In general, there are two forms of urban land conversion: urban land development and urban land redevelopment [5–6]. Urban land development means that non-urban land at the edge of the urban built-up area is converted into urban land; this can reflect the enhancement of a certain type of urban function at the edge of urban built-up area. Urban land redevelopment means that one type of urban land is converted into other types of urban land within the urban built-up area (referred to as “convert out”). At the same time, other types of urban land may also be converted into this type of urban land (referred to as “convert in”). Clearly, urban land redevelopment can reflect the mutual replacement of urban functions. Overall, the change of urban land is determined by urban land development and urban land redevelopment. Also, urban land development and urban land redevelopment can reflect changes in urban functions. Generally, urban land development increases the area of urban land. However, in the case of urban land redevelopment, because one type of urban land is transformed into other types of urban land, other types of urban land will be transformed into that type of urban land at the same time. Therefore, through the redevelopment of urban land, the area of urban land may either increase, decrease, or not change, leading to uncertain changes in urban functions. Thus, by analyzing the relationship between urban land development and redevelopment, we can evaluate the enhancement and weakening of urban functions, and assess the temporal and spatial characteristics of changes in urban functions, to increase attention in this field. Therefore, the main innovation of this method is to use urban land use conversion to identify the spatial difference of the enhancement and weakening of urban functions, not just the classification of urban functions. Taking Changchun, China as the study area, this paper quantitatively measures the enhancement and weakening of the residential, commercial, public service and industrial functions in the central city of Changchun and their temporal and spatial changes. We chose Changchun as the study object for the following reasons: Changchun is a less developed city. The development and redevelopment of urban land is very active, which is very helpful to the practice of urban land conversion methods. Moreover, this method can also be applied to many less developed cities around the world. The quantitative measurement of urban function is a very important research topic in the field of urban geography and urban planning. We hope that this research can promote quantitative research on changes in the enhancement and weakening of urban functions and enrich the methods of understanding urban functions.

The rest of the paper is organized as follows. Section 2 introduces the study area, data sources and methods. Section 3 describes the results in detail, including the spatial and temporal changes in the enhancement and weakening of residential, commercial, public service and industrial functions. Section 4 discusses the results and summarizes the main conclusions.

## 2. Materials and methods

### 2.1 Study area

Changchun is the capital city of the Jilin Province and the second largest city in northeast China. It has a total area of 20,604 km^2^, and a total population of 7.54 million in 2015 [40]. Changchun’s history of urban construction began in 1800, making it a relatively young city for China. Changchun is treated as a mono-centric city which is reasonable due to the historical development of the city from perhaps as early as the period when Japan occupied the city. The urban planning level of Changchun during the Japanese occupation period is very high, and it is worth our continued attention and research on this city. The urban construction during the Japanese occupation laid a solid foundation for Changchun’s future urban space development. After the establishment of the People’s Republic of China, Changchun’s urban planning continued and improved on this basis. Our research object is the central city, which covers an area of about 612 km^2^ and had a population of 3.48 million in 2015. The area of the central city accounts for only 2.97% of the total area of Changchun, but its population accounts for 46.15%. Therefore, the central city is the veritable core of Changchun.

**Fig 1:**
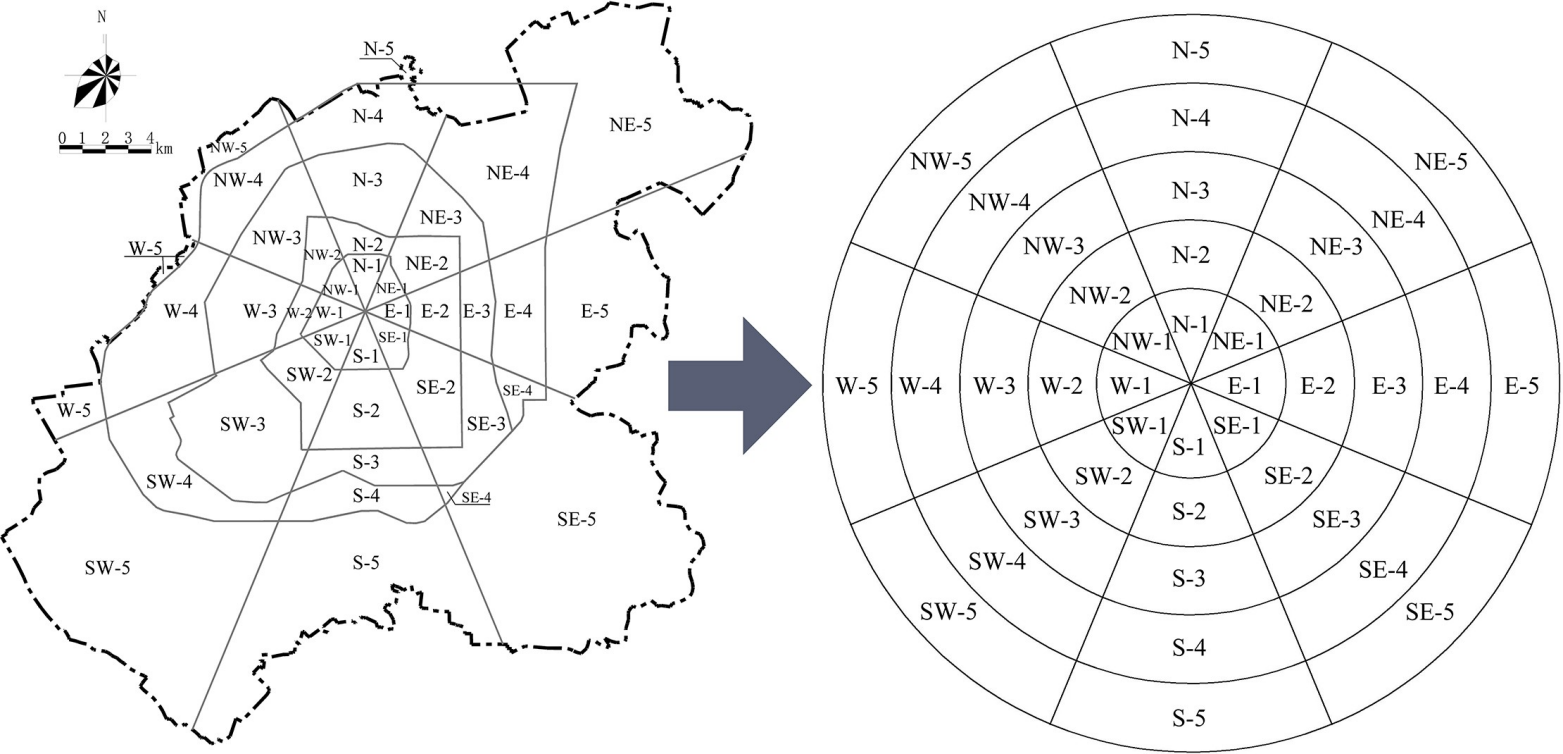
Schematic diagram of the central city of Changchun and its research unit encoding.

Sector analysis and circle analysis are two commonly used spatial analysis methods in urban studies. The former helps us analyze directional features, while the latter helps analyze concentric features. In this study, we combined these two methods. Taking the People’s Square as the center, the central city was divided into eight directions. There are four ring-roads in the central city. From the inside to the outside, there are the first ring-road, the second ring-road, the third ring-road, and the fourth ring-road. Based on the four ring-roads and the eight directions, the central city can be divided into 40 research units. In order to facilitate identification and analysis, we simplified and encoded the 40 research units (Fig 1), which were named according to the method of “direction-zone”. The “direction” represents one of the eight directions, and the “zone” represents the concentric rings divided by the ring-roads. The letters N, NE, E, SE, S, SW, W, and NW represent the eight directions of north, northeast, east, southeast, south, southwest, west, and northwest, respectively, while the numbers 1, 2, 3, 4, 5 represent the area inside the first ring-road, between the first and second ring-road, between the second and third ring-road, between the third and fourth ring-road, and outside the fourth ring-road, respectively. For instance, NE-4 means the area between the third and fourth ring-road in the northeast part of the central city.

### 2.2 Data sources

We used mainly urban land use maps and digital topographic maps of the central city with a scale of 1:10,000, covering the years 2003, 2007, 2011, and 2015. The basic data sources of our study are topographic maps drawn by professional surveyors. In addition, the planners made a detailed urban land use map on the basis of the topographic maps through field visits, and performed annual updates. According to the *Code for Classification of Urban Land Use and Planning Standards of Development Land (GB 50137–2011)*, which was formally issued in 2012, the urban development land in the central city of Changchun can be divided into nine categories: residential land (RL); commercial land (CL); public service land (PL); industrial land (IL); logistics and warehouse land (LWL); road and transport facility land (RTL); municipal utility land (ML); green space and square land (GSL); and special land (SL). Urban land use maps have very detailed land use classifications and include very reliable data; therefore, they are an important basis for our study. Residential land, commercial land, public service land, and industrial land are the four most important types of urban land, accounting for nearly 70% of the total urban development land. For this reason, in this study we focused on the changes in these four types of urban development land. Based on the urban land use maps for 2003, 2007, 2011, and 2015, a database of urban land use layouts of the central city in Changchun was established. In 2003, 2007, 2011 and 2015, the area of residential land is 66.03 km^2^, 75.93 km^2^, 97.38 km^2^, 108.21 km^2^, respectively. The area of public service land is 27.25 km^2^, 36.80 km^2^, 32.27 km^2^, 36.33 km^2^, respectively. The area of commercial land is 8.81 km^2^, 12.56 km^2^, 13.78 km^2^, 18.48 km^2^, respectively. The area of industrial land is 54.64 km^2^, 64.34 km^2^, 71.92 km^2^, 75.10 km^2^, respectively.

### 2.3 Methods

#### 2.3.1 Urban land development

The overlay analysis was used to identify the development of urban land. In our work, the vector data of urban land use map was used to analyze the development and redevelopment of urban land. With the help of the AutoCAD 2008 and ArcGIS 9.3 software platforms, using ArcToolbox in ArcMap, urban land use maps of different years were superimposed to obtain the spatial layout of the development of different types of urban land. Then, the spatial distribution maps of residential, commercial, public service, and industrial land development in the three phases of 2003–2007, 2007–2011, and 2011–2015 were drawn. The area of urban land development of each research unit was obtained according to the 40 research units identified. Urban land development is the conversion of non-urban land into urban land; therefore, the area of urban land development is greater than, or equal to, zero.

#### 2.3.2 Urban land redevelopment

The transition matrix method was used to analyze the redevelopment of urban land [41]. Using the urban land layout database and transition matrix, as well as the ArcGIS 9.3 software platform, the spatial layout of urban land redevelopment was obtained, including “convert-in” and “convert-out”. Then, the spatial distribution maps for the “convert-in” and “convert-out” of residential, commercial, public service, and industrial land in the three phases (2003–2007, 2007–2011, and 2011–2015) were drawn. Similarly, we obtained the “convert-in” and “convert-out” areas of urban land in the 40 research units. By adding the “convert-in” and “convert-out” areas, we obtained the area of urban land redevelopment in each research unit. When the area of urban land redevelopment is less than zero, this indicates that urban land has been reduced through urban land redevelopment; on the contrary, when it is greater than zero, this indicates that urban land has been increased through urban land redevelopment; finally, when it is equal to zero, this shows that, although the redevelopment of urban land has occurred, it has not caused changes in the area of urban land.

#### 2.3.3 Changes in urban functions based on urban land use conversion

As mentioned above in Section 2.3.1 and 2.3.2, the area of urban land development may be greater than, or equal to zero, and the area of urban land redevelopment may be greater than, less than, or equal to zero. Thus, the change in the area of urban land use conversion caused by urban land development and redevelopment may also be uncertain. According to the relationship between urban land development and redevelopment, shown in Table 1, in theory the change status of urban function can be divided into six categories: weak enhancement; strong enhancement; weak weakening; strong weakening; stabilization; and counterbalance stabilization. As the status of counterbalance stabilization did not appear in our empirical research, it was not further discussed.

**Table 1:** Change status of urban function caused by urban land use conversion.

Urban land redevelopment	Urban land development	Urban land use conversion	Change of urban function
UL_R_>0	UL_D_ = 0	UL_C_>0	Weak enhancement
	UL_D_>0	UL_C_>0	Strong enhancement
UL_R_ = 0	UL_D_ = 0	UL_C_ = 0	Stabilization
	UL_D_>0	UL_C_>0	Weak enhancement
UL_R_<0	UL_D_ = 0	UL_C_<0	Weak weakening
	UL_D_>0	UL_C_<0	Strong weakening
		UL_C_ = 0	Counterbalance stabilization
		UL_C_>0	Strong enhancement

UL_R_ indicates the area change in urban land redevelopment; UL_D_ indicates the area change in urban land development; and UL_C_ indicates the area change in urban land caused by urban land development and redevelopment.

## 3. Results

### 3.1 Spatiotemporal differences in the enhancement and weakening of the residential function

**Fig 2:**
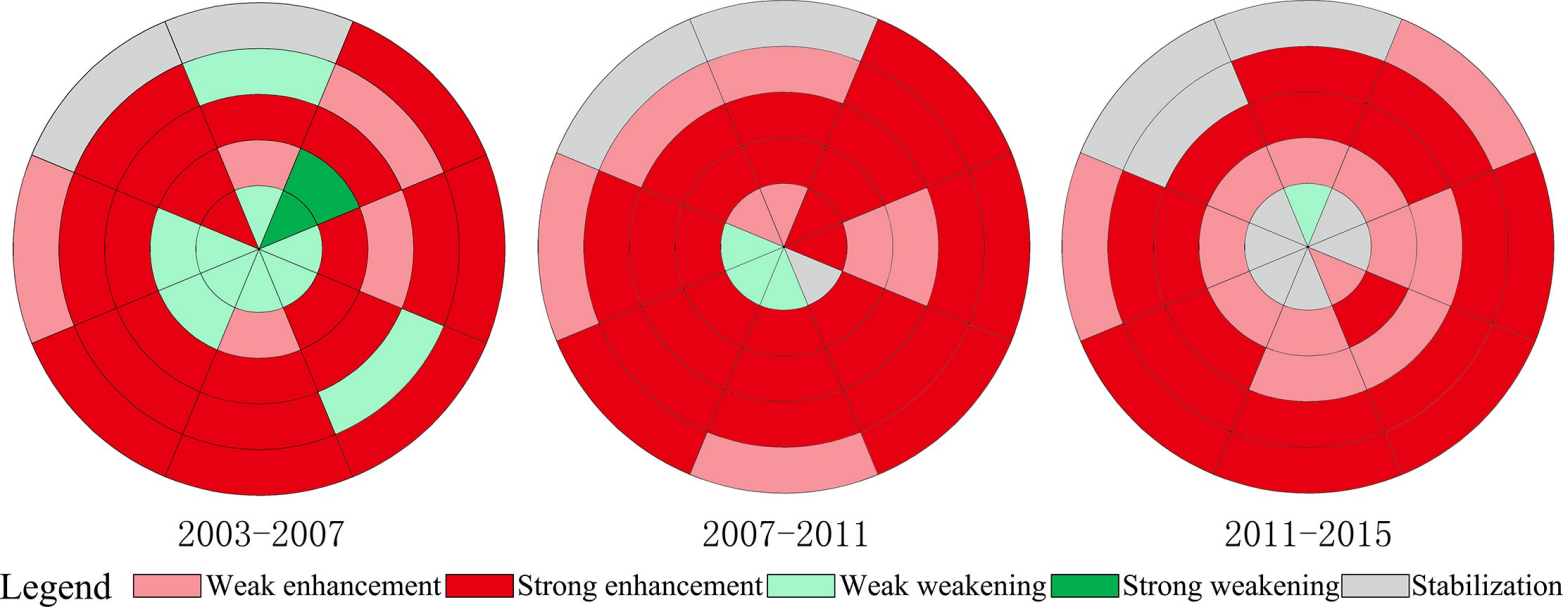
Spatiotemporal change of the residential function in the central city of Changchun.

Looking at the spatiotemporal change of the residential function, it can be seen that it was mainly enhanced, with an average of 30 research units in each of the three temporal phases (Fig 2). The research units of enhanced residential function were distributed in various directions and circles, and the number of research units with strong enhancement of residential function was far greater than that with weak enhancement. It can also be seen that the development and redevelopment of residential land have jointly promoted the enhancement of the residential function. The residential function in the inner circle was enhanced by residential land redevelopment, while in the outer circle, the residential function was enhanced by residential land development and redevelopment. The research units with a strong enhancement of the residential function were constantly moving outwards, and the outer circle has gradually become a key area for housing development. Hence, there was a clear trend of suburbanization of residential functions.

The weakening of the residential function was not a major trend. The number of research units with weakened residential function decreased from 12 in 2003–2007 to 1 in 2011–2015 (Fig 2). The research units with weakened residential function were mainly distributed within the second ring-road, especially within the first ring-road. Our research found that the improvement of commercial and public service functions within the second ring-road was the main reason for the weakening of the residential function. In other words, commercial and public service land replaced a large number of residential lands to improve urban function and the quality of life of residents. Changchun is a less developed city, and its functions are gradually improving.

The area with stable residential function was constantly expanding. The number of research units with stable residential function increased from 2 in 2003–2007 to 9 in 2011–2015 (Fig 2). The research units with stable residential function showed clear spatial characteristics, being distributed within the first ring-road and outside the fourth-ring road. Within the first ring-road (i.e., NE-1, E-1, S-1, SW-1, W-1, and NW-1), urban construction has become increasingly mature and stable, and the development and redevelopment of urban land has been completed in stages. As a result, residential function has become increasingly stable. In the NW and N directions outside the fourth ring-road, the residential function was stable from 2003 to 2015. This is related to the government’s urban planning and the city’s development strategy. The Changchun Municipal Government has not directed urban space development along the NW and N directions, which is also reflected in the subsequent research on commercial, public service, and industrial functions.

### 3.2 Spatiotemporal differences in the enhancement and weakening of the commercial function

**Fig 3:**
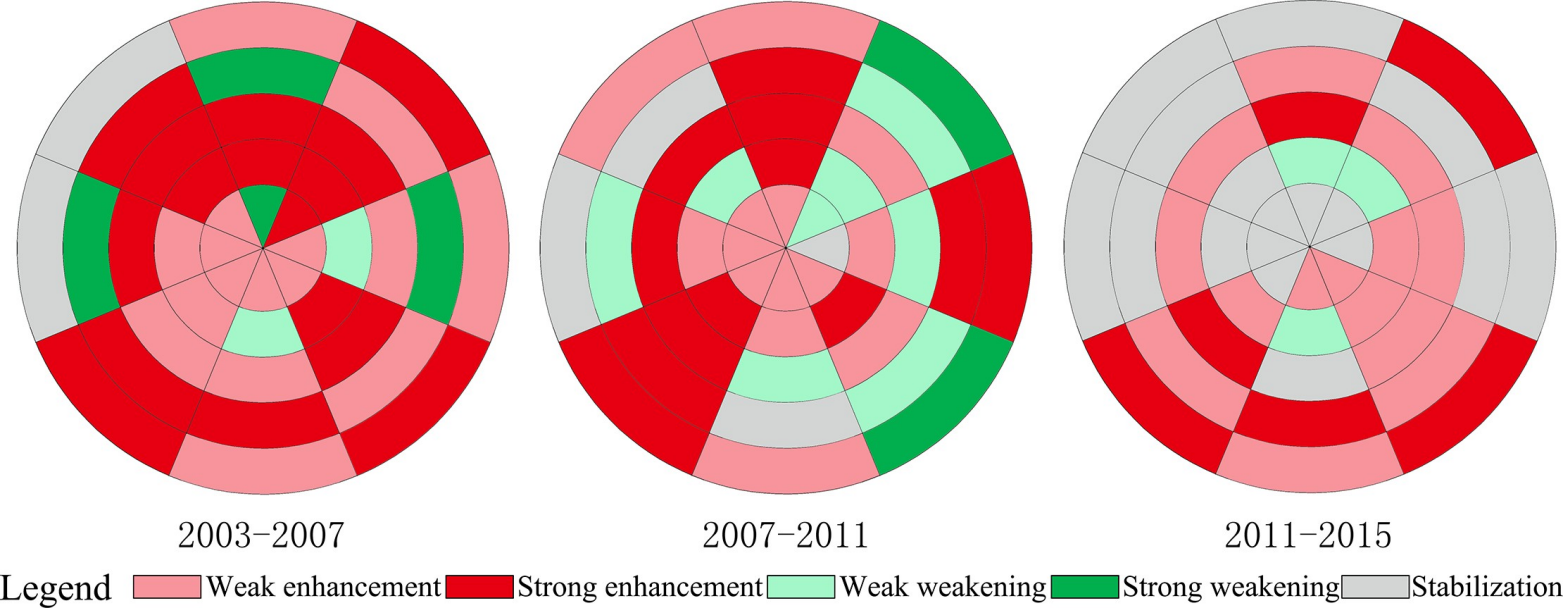
Spatiotemporal change of the commercial function in the central city of Changchun.

From 2003 to 2015, the commercial function primarily enhanced, with an average of nearly 27 research units at each phase (Fig 3). In fact, as mentioned in Section 1, Changchun is a less developed city and is undergoing rapid development, and the commercial function is also in the process of continuous improvement. However, a decline has been observed in the number of research units with enhanced commercial function, from 32 in 2003–2007 to 28 in 2007–2011 and 20 in 2011–2015 (Fig 3). This indicates that the commercial function of the central city has been greatly improved. Moreover, the number of research units with a strong enhancement of the commercial function decreased more significantly, from 16 to 6 between 2003 and 2015. The proportion of research units with a weak enhancement increased from 50% to 70% in 2003–2015. This shows that the enhancement of the commercial function was mainly driven by either the development, or the redevelopment of commercial land. In fact, within the third ring-road, the redevelopment of commercial land led to the enhancement of the commercial function; while outside the third ring-road, the development of commercial land led to the enhancement of commercial function.

The number of research units with weakened commercial function first increased, from 6 in 2003–2007 to 10 in 2007–2011, and then decreased to 3 in 2011–2015. Generally, commercial land has the highest price, and it is difficult to replace it with other types of urban lands. Through our field visits, we discovered the reason for the weakening of the commercial function. Since Changchun is in the process of rapid development, there are a large number of low-level commercial facilities in the city. With the maturing of urban development, these low-level commercial facilities are constantly being replaced by other urban functions, resulting in a weakening of the commercial function, especially in research units with a strong weakening of commercial functions. During the period 2011–2015, the commercial function weakened only in 3 research units, indicating that low-level commercial facilities have gradually been replaced.

The number of research units with a stable commercial function increased from 2 in 2003–2007 to 4 in 2007–2011 and to 17 in 2011–2015 (Fig 3). Similar to the residential function, the commercial function was stable for two reasons. The first reason is related to the strategy of urban development. In the non-main direction of urban development (W-4, W-5, NW-4, NW-5, and N-5), there was no change in urban functions, and the commercial function tended to stabilize. The second reason is related to the maturity of urban development. In the core area of the central city, urban construction was gradually saturated and urban functions tended to stabilize, leading to the stabilization of commercial function. As shown in Fig 3, the commercial function within the first ring-road was stable during the period 2011–2015.

### 3.3 Spatiotemporal differences in the enhancement and weakening of the public service function

**Fig 4:**
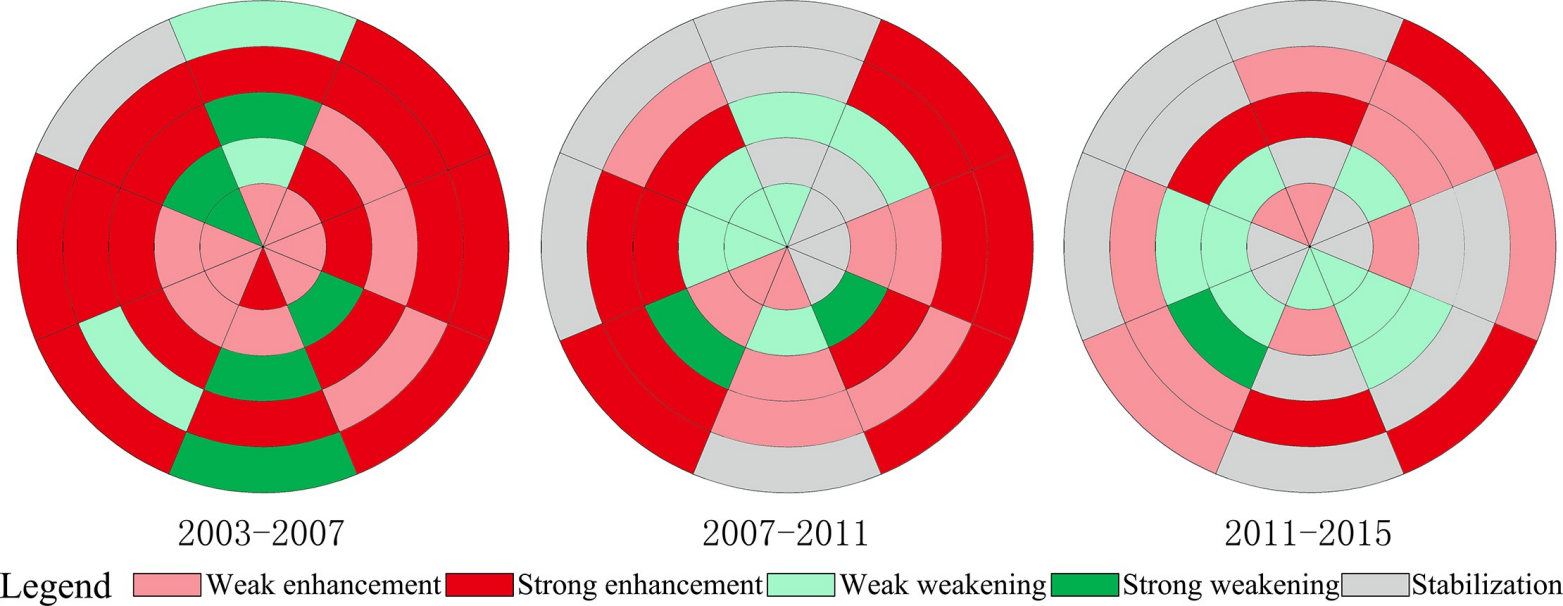
Spatiotemporal change of the public service function in the central city of Changchun.

On the whole, the public service function improved during the period 2003–2015, with an average of 22 research units in each phase. The number of research units with enhanced public service function decreased from 30 in 2003–2007 to 20 in 2007–2011 and to 16 in 2011–2015 (Fig 4). The area of enhanced public service function has been shrinking. The research units with a strong enhancement of the public service function were mainly distributed outside the third ring-road. The development and redevelopment of public service land outside the third ring-road has led to a strong enhancement of the public service function. In all three phases, the public service function of the research units SE-3, SE-5, NE-5, and NW-3 has been in a state of strong enhancement. The expansion of public service land, that is, the new construction of public service facilities such as Universities and exhibitions, has led to the enhancement of the public service function of these research units.

The number of research units with weakened public service function has not changed much, from 9 in the first phase to 10 in the following two phases (Fig 4). In China, public service land is of public interest, and is allocated by the government to provide public services for residents, including education, medical, cultural, entertainment, and government facilities. Under normal circumstances, public service land will not be replaced by other types of urban land. However, public service land in the central city of Changchun has been largely replaced, especially within the third ring-road. The weakening of the public service function was driven mainly by the redevelopment of public service land. On the one hand, some public service land was converted into commercial and residential land to promote the improvement of urban functions. On the other hand, the relocation of administrative and educational facilities led to the weakening of the public service function in the inner city, and especially to a strong weakening of the public service function.

The number of research units with stable public service function increased from 1 in 2003–2007 to 10 in 2007–2011 and 14 in 2011–2015 (Fig 4). Within the second ring-road, the reason for the stability of the public service function is the maturity of urban construction and the improvement of various urban functions. Outside the second ring-road, two reasons led to the stability of the public service function. The first reason is that the development of the public service function lags behind other urban functions, and it will be enhanced in the future, such as in the case of research units E-3, E-4, S-3, S-5, SE-4, and W-5. The second reason is that the public service function is not along the main directions of urban space expansion and is ignored by urban development, such as in the case of research units N-4, N-5, and NW-5.

### 3.4 Spatiotemporal differences in the enhancement and weakening of the industrial function

**Fig 5:**
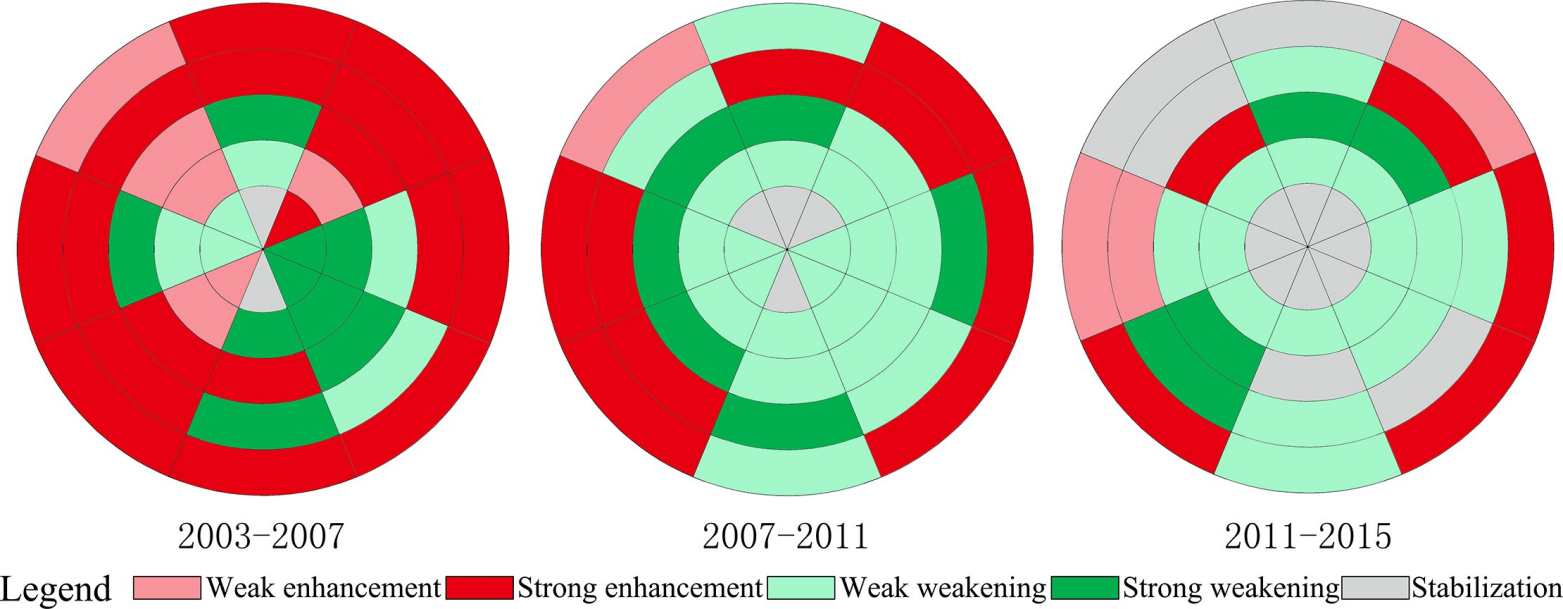
Spatiotemporal change of the industrial function in the central city of Changchun.

The number of research units with enhanced industrial function decreased from 23 in 2003–2007 to 10 in 2007–2011 and 8 in 2011–2015 (Fig 5). Moreover, the research units with industrial function enhancement were mainly distributed outside the third ring-road. The industrial function gradually diffused to the periphery through the development of industrial land. It mainly improved during the period 2003–2007, while it weakened in the following two phases. The future of industrial land is not in the central city, as it will migrate to further suburbs, leading to the suburbanization of industry, while industrial land in the inner city is gradually being replaced by other types of urban land.

The weakening trend of the industrial function is evident, with an average of 23 research units in each stage. In addition, the number of research units with weakened industrial function increased from 15 in 2003–2007 to 26 in 2007–2011 and 29 in 2011–2015 (Fig 5). Due to historical reasons, a large quantity of industrial land in Chinese cities is located in the inner city. With the expansion of urban space, the contradiction between industrial land and other types of urban land has become increasingly prominent, and led to the constant relocation of industrial land. A large amount of industrial land in the inner city has been replaced by other types of urban land, resulting in the continued weakening of the industrial function. In addition, research units with weakened industrial function have been moving outwards, indicating that industrial land in the inner city has gradually been replaced. In addition, research units with weakened industrial function were widely distributed in the last two phases, indicating that industrial land in the central city was widely replaced and continued to migrate to the periphery.

The number of research units with stable industrial function increased from 2 in 2003–2007 to 4 in 2007–2011 and 13 in 2011–2015 (Fig 5). In the inner city, especially within the first ring-road, industrial land has been completely replaced, and the industrial function tended to be stable. Within the first ring-road, the industrial function of 2, 4, and 8 research units in the three different phases has stabilized, indicating that the de-industrialization process has been completed within the first ring-road, and industrial land will continue to shift to the periphery in the future. Like for other urban functions, the main reason for the stability of the industrial function in the research units N-5, NW-4, and NW-5 is that they are not located along the main directions of urban space expansion.

## 4. Discussion and conclusions

In previous studies, scholars usually considered cities as a “point” at the regional level to explore changes in their urban functions. Based on the regional perspective, the functions of tourism and transportation in cities are widely discussed [42–43].This study regarded cities as a “plane” to analyze the changes in various urban functions within them. This study reflected changes in urban functions through changes in urban land use. Urban land use conversion includes urban land development and redevelopment, which can help analyze the inherent characteristics of urban function changes. Based on this, we quantitatively identified the enhancement and weakening of residential, commercial, public service, and industrial functions in the central city of Changchun, and analyzed their spatiotemporal changes. Moreover, the change state of urban function enhancement and weakening could be divided into six categories: weak enhancement; strong enhancement; weak weakening; strong weakening; stabilization; and counterbalance stabilization. With the help of urban ring-roads and directions, we also analyzed the characteristics of the spatiotemporal changes in the enhancement and weakening of urban functions.

With the advancement of technology, tax OD trips data[1], POI data[2], mobile phone data[7], social media data[7] and vehicle trajectory data[32] and other big data[33–37] are used in the identification of urban functions. Although scholars can quickly and accurately identify the types of urban functions, it is difficult to identify the enhancement and weakening of urban functions. This research shows that urban land use conversion is an effective method to quantitatively identify changes in urban functions. Our research can identify not only the types of urban functions, but also the enhancement and weakening of urban functions, making up for the vacancy in this field. With the diversification of research methods, urban land use data is becoming more and more accessible. Therefore, the research method in this paper can be widely extended to other cities, especially to study the rapid changes of urban functions in less developed cities.

The enhancement and weakening of residential, commercial, public service, and industrial functions present similar characteristics. In general, residential, commercial, and public service functions of the central city of Changchun mainly enhanced, while the industrial function mainly weakened. Less developed cities in China are undergoing a process of suppression of the secondary sector and development of the tertiary sector, i.e., deindustrialization. Therefore, the industrial land in the inner city of Changchun is gradually being replaced by other types of urban land such as residential, commercial, and public service land, and is moving to the periphery. As a result, the residential, commercial, and public service functions of the central city are significantly enhanced, while the industrial function is significantly weakened. In addition, these four types of urban function within the first ring-road were basically stable, and there was almost no urban land development and redevelopment. With the improvement of various urban facilities, urban construction within the first ring-road has gradually matured, leading to the stabilization of urban functions. In relation to the changes of the four types of urban function, there is another stable situation: stagnant stabilization. Urban spatial expansion follows different key directions at different stages of development. Urban spatial expansion in Changchun did not develop along the NW and N directions in the three phases investigated. Thus, residential, commercial, public service, and industrial functions tended to stabilize in the NW and N directions for a long period of time, especially in the case of research units NW-5 and N-5.

The enhancement and weakening of residential, commercial, public service, and industrial functions also exhibited different spatial characteristics. Compared with the other three urban functions, the number of research units with weakened residential function was the lowest, indicating that the enhancement of the residential function was the most significant during the three phases investigated. The characteristics of spatiotemporal changes in residential, commercial, and public service functions indicate that the residential function dominated the urban spatial development in Changchun during the phase of transformation and development. The public service function is an important way to assist residential development and to promote the rapid development of urban space. Therefore, the relocation of the municipal government and the construction of the University town guided the development of residential and urban space. As a result, the public service function has also shown a relatively clear trend of relocation. However, the development and redevelopment of the commercial function exhibited different characteristics. Unlike public service facilities, commercial facilities are not public welfare. Following market rules, the development of commercial facilities often lags behind the construction of residential and public service facilities. In the process of development of residential and public service spaces, there was no population gathering, so there was a certain degree of lag in the development and redevelopment of commercial land. Unlike for the spatial changes of the other three types of urban function, the industrial function showed a clear suburbanization due to de-industrialization.

This study confirmed the effectiveness of the urban land use conversion method, demonstrating that through the analysis of the spatiotemporal changes of residential, commercial, public service, and industrial functions. It is difficult to measure the enhancement and weakening of various urban functions within a city. Based on urban land use conversion, we analyzed the inherent characteristics of urban land use change. Through urban land development and redevelopment, we quantitatively analyzed the characteristics of the spatiotemporal changes in the enhancement and weakening of urban functions. Clearly, interesting results have been achieved. Our research on the enhancement and weakening of urban functions in the central city of Changchun provides theoretical support for the sustainable development of the urban space in the future. On the practical level, the study on the enhancement and weakening of urban functions can provide insight into the internal mechanism of urban spatial structure evolution, and provide references for the optimization of urban spatial structure and the rational development of urban space.

Although land is the carrier of urban functions, there is a certain one-sidedness in quantitatively measuring urban functions only from the perspective of urban land use. In the future, we will consider additional factors to improve the scientificity of the quantitative measurement of urban function enhancement and weakening.
